# BRCA1 and BRCA2 as molecular targets for phytochemicals indole-3-carbinol and genistein in breast and prostate cancer cells

**DOI:** 10.1038/sj.bjc.6602935

**Published:** 2006-01-24

**Authors:** S Fan, Q Meng, K Auborn, T Carter, E M Rosen

**Affiliations:** 1 Department of Oncology, Lombardi Comprehensive Cancer Center, Georgetown University, 3970 Reservoir Road, NW, Washington, DC 20057-1469, USA; 2 Department of Otolaryngology, North Shore-Long Island Jewish Research Institute, BoasMarks Biomedical Science Research Center, 350 Community Drive, Manhasset, New York 11030, USA

**Keywords:** indole-3-carbinol (I3C), genistein, chemoprevention, BRCA1, BRCA2, EMR, endoplasmic reticulum stress response

## Abstract

Indole-3-carbinol (I3C) and genistein are naturally occurring chemicals derived from cruciferous vegetables and soy, respectively, with potential cancer prevention activity for hormone-responsive tumours (e.g., breast and prostate cancers). Previously, we showed that I3C induces BRCA1 expression and that both I3C and BRCA1 inhibit oestrogen (E2)-stimulated oestrogen receptor (ER-*α*) activity in human breast cancer cells. We now report that both I3C and genistein induce the expression of both breast cancer susceptibility genes (BRCA1 and BRCA2) in breast (MCF-7 and T47D) and prostate (DU-145 and LNCaP) cancer cell types, in a time- and dose-dependent fashion. Induction of the BRCA genes occurred at low doses of I3C (20 *μ*M) and genistein (0.5–1.0 *μ*M), suggesting potential relevance to cancer prevention. A combination of I3C and genistein gave greater than expected induction of BRCA expression. Studies using small interfering RNAs (siRNAs) and BRCA expression vectors suggest that the phytochemical induction of BRCA2 is due, in part, to BRCA1. Functional studies suggest that I3C-mediated cytoxicity is, in part, dependent upon BRCA1 and BRCA2. Inhibition of E2-stimulated ER-*α* activity by I3C and genistein was dependent upon BRCA1; and inhibition of ligand-inducible androgen receptor (AR) activity by I3C and genistein was partially reversed by BRCA1-siRNA. Finally, we provide evidence suggesting that the phytochemical induction of BRCA1 expression is due, in part, to endoplasmic reticulum stress response signalling. These findings suggest that the BRCA genes are molecular targets for some of the activities of I3C and genistein.

Indole-3-carbinol (I3C) is a phytochemical found in cruciferous vegetables, such as cabbage and cauliflower. Epidemiologic studies suggest a correlation between a diet high in such vegetables and reduced breast cancer rate (Graham *et al*, 1982). In animal studies, a diet high in these vegetables inhibited chemical carcinogen-induced tumours; and dietary supplementation with I3C could prevent oestrogen-dependent tumours (breast, cervical, endometrial cancers) (Bradlow *et al*, 1991; Kojima *et al*, 1994; Jin *et al*, 1999). In clinical studies, I3C caused regression of cervical intraepithelial neoplasia (Bell *et al*, 2000) and regression or a decreased growth rate of recurrent laryngeal polyps (Rosen *et al*, 1998). Indole-3-carbinol anticancer activity is attributed, in part, to its anti-oestrogenic activity: it stimulates 2-hydroxylation and inhibits 16*α*-hydroxylation of oestrone, leading to inactive oestrone metabolites (Michnovicz and Bradlow, 1990). Indole-3-carbinol may also interact directly with the oestrogen receptor (ER-*α*) and inhibit its activity (Jin *et al*, 1999); and it cooperates with Tamoxifen to inhibit breast cancer cell proliferation (Cover *et al*, 1999).

Indole-3-carbinol also exerts oestrogen-independent actions. It can inhibit cell cycle progression, induce apoptosis, and inhibit tumour invasion and metastasis, even in ER-*α*-negative cells (Meng *et al*, 2000a, 2001; Bonnesen *et al*, 2001; Chen *et al*, 2001). Indole-3-carbinol downregulates expression of a G1 cyclin-dependent kinase (CDK6) and upregulates a CDK inhibitor (p21^Cip1^) (Cover *et al*, 1998). The major active metabolite of I3C, diindolylmethane (DIM), induces expression of GADD45*α*, a DNA damage-responsive gene and putative tumour suppressor (Carter *et al*, 2002). Indole-3-carbinol can activate two pathways linked to cancer prevention: (1) aryl hydrocarbon receptor (AhR) signalling (which leads to expression of phase I enzymes (e.g., CYP1A1) via the xenobiotic response element) and (2) antioxidant/electrophilic response element signalling (resulting in expression of phase II detoxifying enzymes: e.g., oxido-reductases and glutathione-*S*-transferases) (Chen *et al*, 1998; Hayes and McMahon, 2001; Kwak *et al*, 2002). Thus, the ability of I3C to induce enzymes that metabolise genotoxic agents may contribute to cancer prevention.

The ability of I3C and DIM to inhibit growth of human prostate cancer cells has raised interest in I3C for prostate cancer prevention. Thus, DIM inhibited dihydrotestosterone (DHT)-stimulated cell proliferation and DHT-induced activation of the prostate-specific antigen promoter in LNCaP cells, by acting as an AR antagonist (Le *et al*, 2003). Indole-3-carbinol induced growth arrest by upregulation of G1 cell cycle inhibitors (p21^Cip1^ and p27^Kip1^) in androgen-independent PC-3 prostate cancer cells (Chinni *et al*, 2001). Indole-3-carbinol and DIM can induce p53-independent apoptosis in prostate cancer cells, in part, due to inhibition of the NF-*κ*B and c-Akt signalling pathways (Chinni *et al*, 2001; Nacheson-Kedmi *et al*, 2003).

We have shown that I3C upregulates expression of the breast cancer susceptibility gene-1 (BRCA1) (Meng *et al*, 2000a 2000b, 2001; Carter *et al*, 2002). Here, we report that: (1) both I3C and genistein, a soy isoflavone with cancer preventive activity for prostate cancer and other tumour types, upregulate both BRCA1 and BRCA2; (2) the BRCA genes contribute to some functional activities of I3C and genistein; and (3) BRCA induction may be due, in part, to stimulation of endoplasmic reticulum stress signalling. These findings have implications for understanding the mechanism(s) of action of these phytochemical cancer prevention agents.

## MATERIALS AND METHODS

### Sources of reagents

Indole-3-carbinol and genistein were obtained from the Sigma Chemical Co. (St Louis, MO, USA) and dissolved in a small amount of dimethylsulphoxide (DMSO) prior to dilution in cell culture medium. 17*β*-Estradiol and DHT were also purchased from Sigma. Thapsigargin and tunicamycin were obtained from Sigma, dissolved in DMSO, and stored at −20°C.

### Cell lines and culture

Human breast (MCF-7 and T47D) and prostate (DU-145, PC-3, and LNCaP) cancer cells were obtained from the American Type Culture Collection (Manassas, VA, USA). All cell lines except LNCaP were grown in Dulbecco's modified Eagle's medium (DMEM) plus 5% (MCF-7, DU-145, and PC-3) or 10% (T47D) (v v^−1^) fetal calf serum, L-glutamine (5 mM), nonessential amino acids (5 mM), penicillin (100 U ml^−1^), and streptomycin (100 *μ*g ml^−1^) (BioWhittaker, Walkersville, MD, USA). LNCaP cells were grown in RPMI 1640 medium plus 10% fetal calf serum and the above additives. Standard cell culture methodology was employed (Fan *et al*, 1998, 2001a, 2001b).

### Expression vectors and reporter plasmids

Wild-type BRCA1 (wtBRCA1) cDNA, cloned into the pcDNA3 expression vector (Invitrogen), was described earlier (Fan *et al*, 1998). To express BRCA2, a full-length human BRCA2 cDNA (provided by Dr Qingshen Gao, New England Medical Center, Boston, MA, USA) was cloned into the pCMV-Tag2B vector (Stratagene). The human ER-*α* expression vector and the oestrogen-responsive reporter ERE-TK-Luc were described before (Fan *et al*, 1999b, 2001a). Wild-type AR expression vector pSG5-AR and the androgen-responsive reporter plasmid ARE-TK-Luc were provided by Dr Chawshang Chang (University of Rochester, Rochester, NY, USA). The ARE-TK-Luc reporter contains an androgen-response element upstream of a minimal thymidine kinase (TK) promoter driving the luciferase gene.

Luciferase reporters driven by the wild-type endoplasmic reticulum stress-response element (ERSEwt-Luc), a mutant ERSE (ERSEmut-Luc, a negative control), and the ERSE-II element (ERSEII3x-Luc) in the pGL3 vector were generously provided by Dr Kazutoshi Mori (HSP Research Institute, Kyoto Research Park, Kyoto, Japan) (Yoshida *et al*, 1998; Yamamoto *et al*, 2004). A CHOP (C/EBP homologous protein) promoter-luciferase reporter in the pGL3 vector (CHOP-Luc) was provided by Dr Pierre Fafournoux (Unite de Nutrition et Metabolisme Proteique, INRA de Theix, Saint Genes Champanelle, France) (Bruhat *et al*, 2000). A dominant negative (DN) PERK expression vector in the pcDNA3 vector (DN-PERK) was kindly provided by Dr Annette C Dolphin (University College London, London, UK) (Page *et al*, 2004). The DN-ATF4 expression vector was provided by Dr Steve F Abcouwer (University of New Mexico School of Medicine, Albuquerque, NM, USA) (Roybal *et al*, 2004), while the DN-IRE1 expression vector was provided by Dr David Ron (Skirball Institute of Biomolecular Medicine, New York University School of Medicine, New York, NY, USA) (Wang *et al*, 1998).

### Cell viability (MTT) assays

Cell viability was determined following the treatments indicated in the figure legends, as described earlier (Fan *et al*, 1998, 1999a). For siRNA treatments, subconfluent proliferating cells in 96-well dishes were transfected with BRCA1 or BRCA2 siRNA (50 nM × 72 h; see below), or mock-treated (transfection reagent only); exposed to different doses of I3C (or vehicle (DMSO) only) for 24 h; and then assayed for MTT dye reduction. For overexpression experiments, the cells were transfected overnight with wtBRCA1 or wtBRCA2 expression vectors (0.06 *μ*g plasmid DNA per well), washed, and postincubated for another 24 h to allow gene expression, prior to exposure to I3C. Cell viability was expressed relative to control cells treated with DMSO only (0 I3C) as mean±s.e.m. of 10 replicate wells.

### Knockdown of BRCA1 or BRCA2 using small interfering RNAs (siRNAs)

The BRCA1-siRNA and control (scrambled sequence) siRNAs were described earlier (Xiong *et al*, 2003). All siRNAs were chemically synthesised by Dharmacon, Inc. For siRNA treatments, subconfluent proliferating cells were transfected with 50 nM of siRNA using the siPORT Amine transfection reagent (Ambion). For both BRCA1 and BRCA2, maximal reduction of protein levels required a 72-h incubation with the siRNA. Prior studies have established that under these conditions, none of the siRNAs caused cytotoxicity, based on cell morphology and MTT assays. The sequences used to synthesise the siRNAs are listed below:

BRCA1-siRNA: 5′-AATGCCAAAGTAGCTAATGTA-3′
Control-siRNA: 5′-CGATAGATACACAGATTGAAT-3′
BRCA2-siRNA: 5′-AACTGAGCAAGCCTCAGTCAA-3′

### Oestrogen receptor-*α* transcriptional activity assay

Oestrogen receptor-*α* activity was measured via transient transfection assays, using an oestrogen-responsive luciferase reporter (Fan *et al*, 1999b, 2001a). Briefly, subconfluent proliferating cells in 24-well dishes were cotransfected overnight with a wild-type ER-*α* expression vector (pSG5-ER-*α*) and the ERE-TK-Luc reporter (0.25 *μ*g of each plasmid per well), in the presence of Lipofectamine™ (Life Technologies, Carlsbad, CA, USA). The cells were washed to remove the excess plasmid and Lipofectamine; allowed to recover for several hours; and postincubated for 24 h in phenol red-free medium containing 2% charcoal-stripped fetal calf serum, with the indicated agents (17-*β*-estradiol (E2, 1 *μ*M) and/or I3C and/or genistein). The doses of each agent are indicated in the figures. Luciferase activity was determined; and the values were expressed relative to the positive control (+E2, no I3C or genistein), as means±s.e.m.'s of three independent experiments, with each assay condition tested in four replicate wells per experiment. To monitor transfection efficiency, cells were cotransfected with control plasmid pRSV-*β*-gal to allow measurement of *β*-galactosidase activity.

### Androgen receptor (AR) transcriptional activity assays

Androgen receptor activity was measured via transient transfection assays, using an androgen-responsive reporter (ARE-TK-Luc). Assays were performed as described above, except that AR-negative PC-3 cells were cotransfected with a wild-type human AR expression vector (pSG5-AR); and the cells were stimulated with DHT (10 nM) for 24 h, again in the absence or presence of the specified doses I3C or genistein. The expression of luciferase activity and the number of replicate assays were the same as described above. Each experiment was repeated at least twice to assure reproducibility.

### mRNA assays

mRNA expression was determined by rigorously controlled semiquantitative RT–PCR assays. For each amplified product, the PCR reaction conditions and cycle numbers were individually adjusted so that all reactions occurred within the linear range of product amplification. The detailed methods for RNA isolation, cDNA synthesis, and RT–PCR analyses for BRCA1, BRCA2, and *β*-actin (control gene) have been described before (Fan *et al*, 1998, 1999a; Xiong *et al*, 2003). For AR, the PCR primers were as follows: 5′-TGTTTTCCCCCTCTTCCTT-3′ (forward) and 5′-TCCTTTTTTCCAGCATAGACC-3′ (reverse). The PCR products were analysed by electrophoresis through 0.8% agarose gels containing 0.1 mg ml^−1^ of ethidium bromide, and the gels were photographed under ultraviolet light. The mRNA levels were quantitated by densitometry of the cDNA bands and expressed relative to *β*-actin. At least two independent experiments were performed for each cell type studied.

### Protein assays

Cell lysates were prepared and Western blotting was performed as described earlier (Fan *et al*, 1998, 1999a). Equal aliquots of total cell protein (50 *μ*g per lane) were electrophoresed on SDS–polyacrylamide gradient gels, transferred to nitrocellulose membranes (Millipore, Billerica, MA, USA), and blotted using these primary antibodies: BRCA1 (C-20, rabbit polyclonal, Santa Cruz Biotechnology, 1 : 200); BRCA2 (C-19, Santa Cruz, 1 : 200); and actin (I-19, goat polyclonal, Santa Cruz, 1 : 500). The proteins bands were visualised using the enhanced chemiluminescence system (Amersham), with coloured markers (BioRad) as molecular size standards. Protein bands were quantitated by densitometry, and the values were expressed relative to actin (control for loading and transfer). At least two independent experiments were performed for each cell type studied.

### Statistical comparisons

Where appropriate, statistical comparisons of the experimental results were made using the two-tailed Student's *t*-test.

## RESULTS

### Indole-3-carbinol upregulates BRCA1 and BRCA2 expression in breast and prostate cancer cells

**Figure 1:**
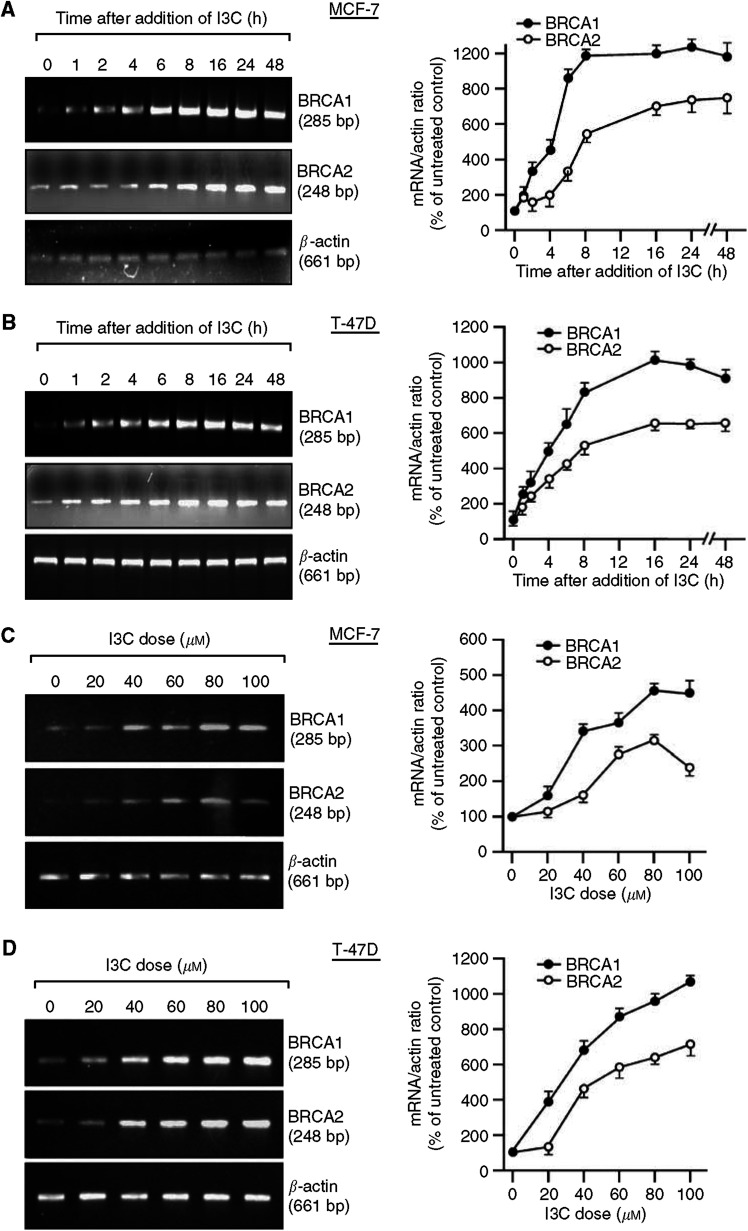
Indole-3-carbinol (I3C) upregulates BRCA1 and BRCA2 mRNA expression in human breast cancer cells in a time- and dose-dependent manner. For time course studies, subconfluent proliferating MCF-7 (**A**) or T47D (**B**) cells were treated with I3C (60 *μ*M) for different times and harvested for mRNA analysis by semiquantitative RT–PCR. For dose–response studies, MCF-7 (**C**) or T47D (**D**) cells were treated with different doses of I3C for 24 h and harvested for mRNA analysis. The PCR bands were quantified by densitometry and expressed relative to the control gene, *β*-actin. The densitometry values are means±s.e.m.'s of at least two independent experiments.

We previously showed that I3C stimulates BRCA1 expression in human cervical and breast cancer cells (Meng *et al*, 2000a, 2000b, 2001; Carter *et al*, 2002). Here, we tested the effect of I3C on BRCA1 and BRCA2 expression in breast and prostate cancer cells. Using rigorously controlled semiquantitative RT–PCR and densitometry analysis, we found that a dose of I3C (60 *μ*M) that causes little or no cytotoxicity caused a time-dependent increase in BRCA1 and BRCA2 mRNA levels in two breast cancer cell lines, MCF-7 and T47D (Figure 1A and B). BRCA1 levels were increased at the earliest time point tested (1 h), reached near maximum by 6–8 h, and were still elevated at 48 h. For T47D, BRCA2 mRNA levels were also elevated by 1–2 h and remained elevated throughout the experiment. MCF-7 cells also showed strong time-dependent induction of BRCA2 by I3C, but consistent increases in BRCA2 mRNA levels were only observed at 6 h and later. Indole-3-carbonil caused dose-dependent induction of BRCA1 and BRCA2 mRNA (measured at 24 h) starting at 20–40 *μ*M (Figure 1C and D). Little or no changes in expression of the control gene (*β*-actin) were evident in any of these experiments.

**Figure 2:**
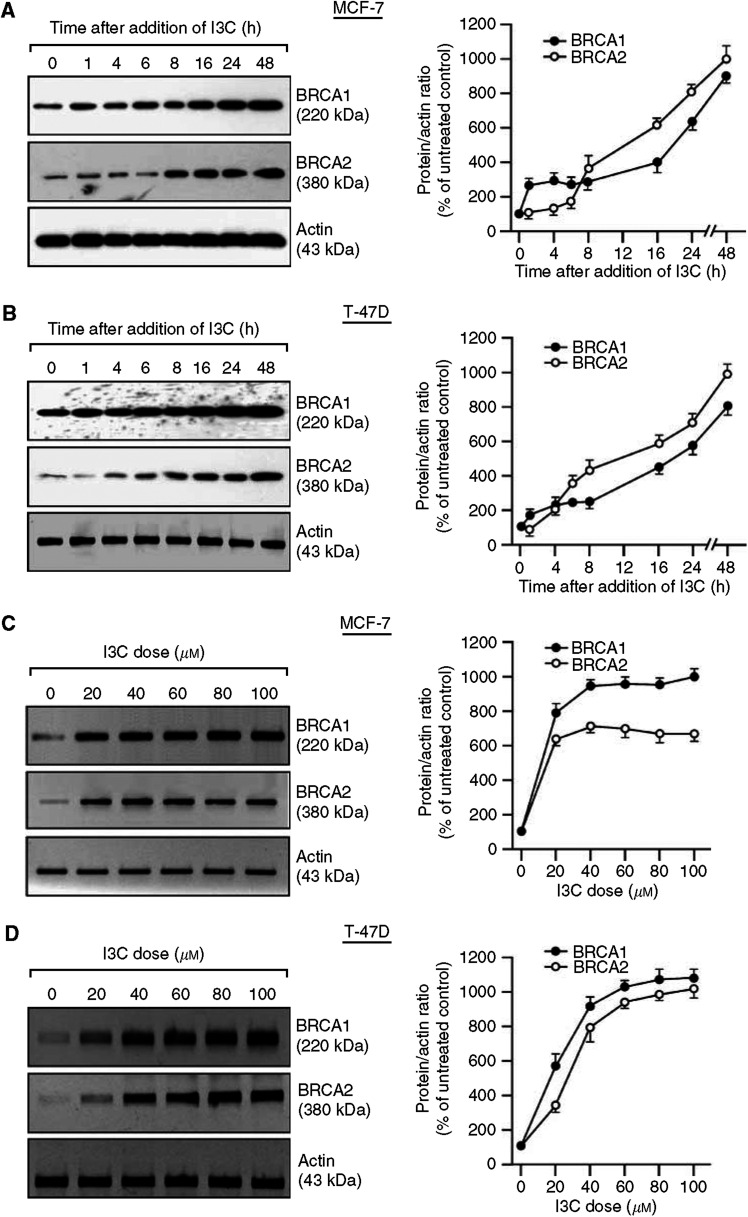

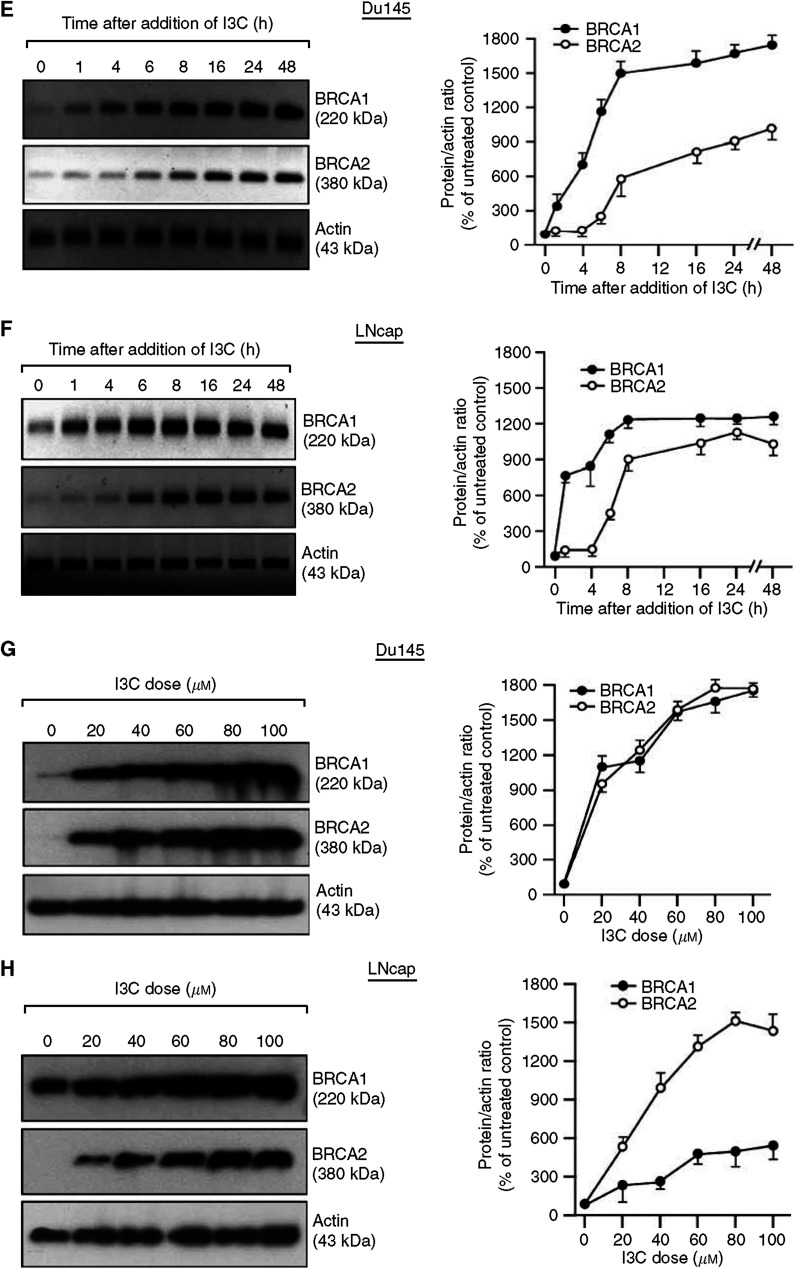
Indole-3-carbinol upregulates BRCA1 and BRCA2 protein levels in human breast and prostate cancer cells in a time- and dose-dependent manner. For time course studies, subconfluent proliferating MCF-7 (**A**), T47D (**B**), DU-145 (**E**), and LNCaP (**F**) cells were treated with I3C (60 *μ*M) for various times and harvested for Western blot analysis to detect the BRCA1, BRCA2, or actin (control for loading and transfer) proteins. For dose–response studies, MCF-7 (**C**), T47D (**D**), DU-145 (**G**), or LNCaP (**H**) cells were treated with the indicated dose of I3C for 24 h and then assayed for BRCA1 and BRCA2 protein expression. The protein bands were quantitated by densitometry and expressed relative to actin. The densitometry values are means±s.e.m.'s of at least two independent experiments.

We tested the effect of I3C on BRCA protein levels by Western blotting with quantitation of the protein bands by densitometry. BRCA protein levels were upregulated by I3C in a time- and dose-dependent fashion in breast (MCF-7 and T47D) and prostate (LNCaP and DU-145) cancer cells (Figure 2). For the breast cancer cells, increased BRCA2 protein levels were first observed after 4–8 h of I3C (60 *μ*M); while increases in BRCA1 levels were detected earlier, but were relatively small in magnitude at early time points (Figure 2A and B). The dose–response studies of MCF-7 and T47D cells revealed significant induction (>2-fold) of BRCA1 and BRCA2 protein levels at the lowest dose of I3C tested, 20 *μ*M (Figure 2C and D). In Figure 2C, the BRCA2 protein induction is not the same as that of the mRNA (Figure 1C). This may be due to inaccuracies in the densitometry quantitation of the RT–PCR (especially when bands are weak), the fact that the protein and mRNA experiments were performed at different times, or the fact that I3C might have an additional effect on protein stability, which we cannot rule out.

Time course studies of prostate cancer cell lines showed a delay in the induction of BRCA2 by I3C (first observed at 6 h) relative to that of BRCA1 (1 h) (Figure 2E and F). As for breast cancer cells, BRCA1 and BRCA2 levels were increased by ⩾2-fold at the lowest dose of I3C (20 *μ*M) (Figure 2G and H). Although there was some variability from cell line to cell line, these studies show reproducible induction of BRCA1 and BRCA2 by I3C, with increases in BRCA1 and BRCA2 protein levels at an I3C dose of 60 *μ*M at 24 h of (5–10)-fold for the breast cancer cell lines and (8–16)-fold for the prostate cancer cell lines. Note: In the studies shown in Figures 1 and 2, the densitometry values are the means of at least two independent experiments.

### Genistein upregulates BRCA1 and BRCA2 expression

**Figure 3:**
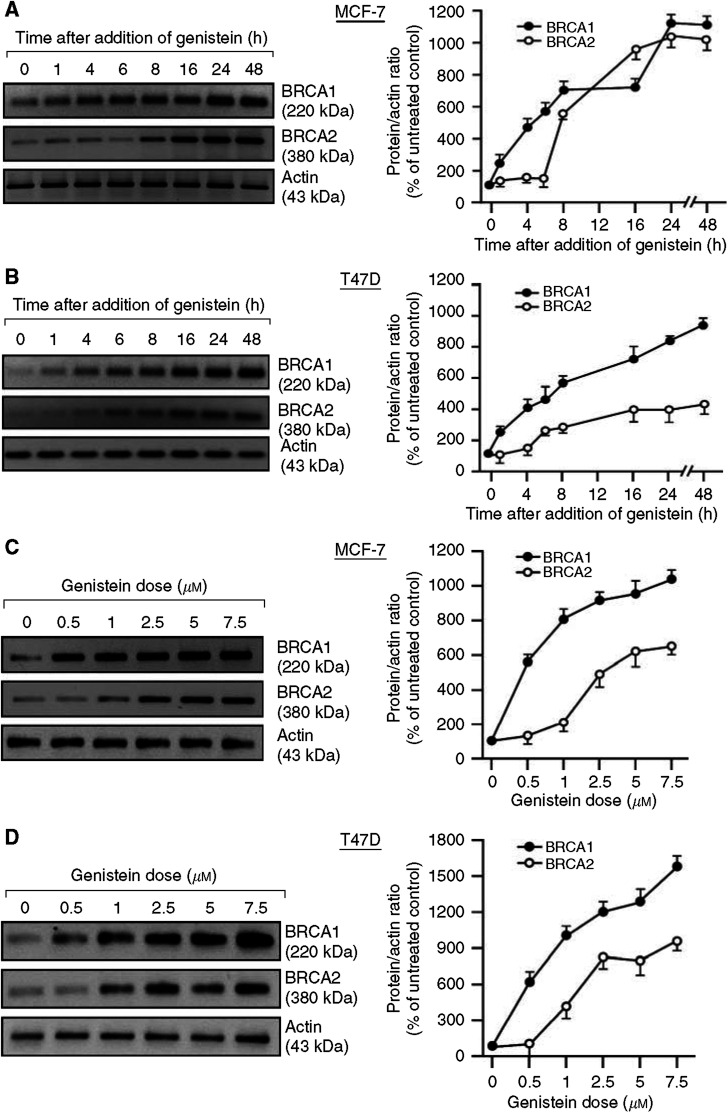

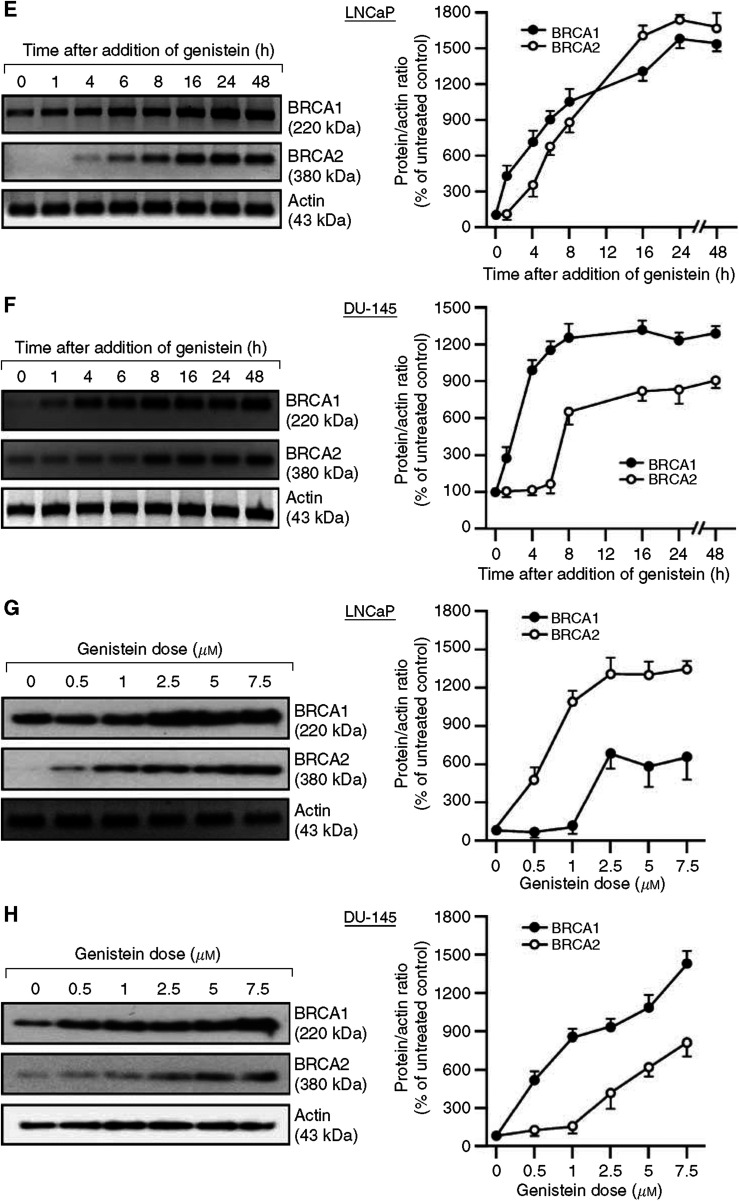
Genistein upregulates BRCA1 and BRCA2 protein levels in breast cancer cells. For time course studies, subconfluent proliferating MCF-7 (**A**), T47D (**B**), DU-145 (**E**), and LNCaP (**F**) cells were treated with genistein (5 *μ*M) for the indicated times and harvested for Western blotting to detect the BRCA1, BRCA2, or actin proteins. For dose–response studies, MCF-7 (**C**), T47D (**D**), DU-145 (**G**), or LNCaP (**H**) cells were treated with the indicated dose of genistein for 24 h and assayed for BRCA1 and BRCA2 protein expression, as described above. The densitometry values represent means±s.e.m.'s of at least two independent experiments.

Genistein is a soy isoflavone that functions, in part, as a phytoestrogen and a phytoandrogen (Wang *et al*, 1996; Maggiolini *et al*, 2002). Since genistein is proposed as a chemoprevention agent for several tumour types (Mentor-Marcel *et al*, 2001; Lamartiniere *et al*, 2002), including breast and prostate cancers, we tested its ability to induce BRCA expression. Genistein caused dose- and time-dependent increases in BRCA1 and BRCA2 protein levels in two oestrogen-responsive breast cancer cell lines (MCF-7 and T47D) (Figure 3D). Induction of BRCA1 occurred after 1 h of genistein exposure (5 *μ*M); while the induction of BRCA2 was delayed until 6–8 h (Figure 3A and B). For both cell lines, increases in BRCA1 levels of >2-fold were observed at the lowest dose of genistein tested (0.5 *μ*M); while similarly robust increases in BRCA2 levels required higher genistein doses (1–2.5 *μ*M) (Figure 3C and D).

Genistein also caused time- and dose-dependent increases in BRCA levels in androgen-sensitive (LNCaP) and insensitive (DU-145) prostate cancer cells. For these cell lines, increases in BRCA1 levels of ⩾ 2-fold occurred at 1 h; while similar increases in BRCA2 levels occurred at 4–8 h (Figure 3E and F). When measured at 24 h, significant increases in BRCA1 levels required doses of 0.5–1.0 *μ*M; while increases in BRCA2 levels required 2.5 *μ*M of genistein (Figure 3G and H). Increases in BRCA levels after exposure to 5 *μ*M of genistein for 24 h ranged (4–12)-fold for breast cancer cells and (5–17)-fold for prostate cancer cells. The densitometry values in Figure 3 are the means of at least two independent experiments.

### Role of BRCA1 in the induction of BRCA2 by I3C and by genistein

**Figure 4:**
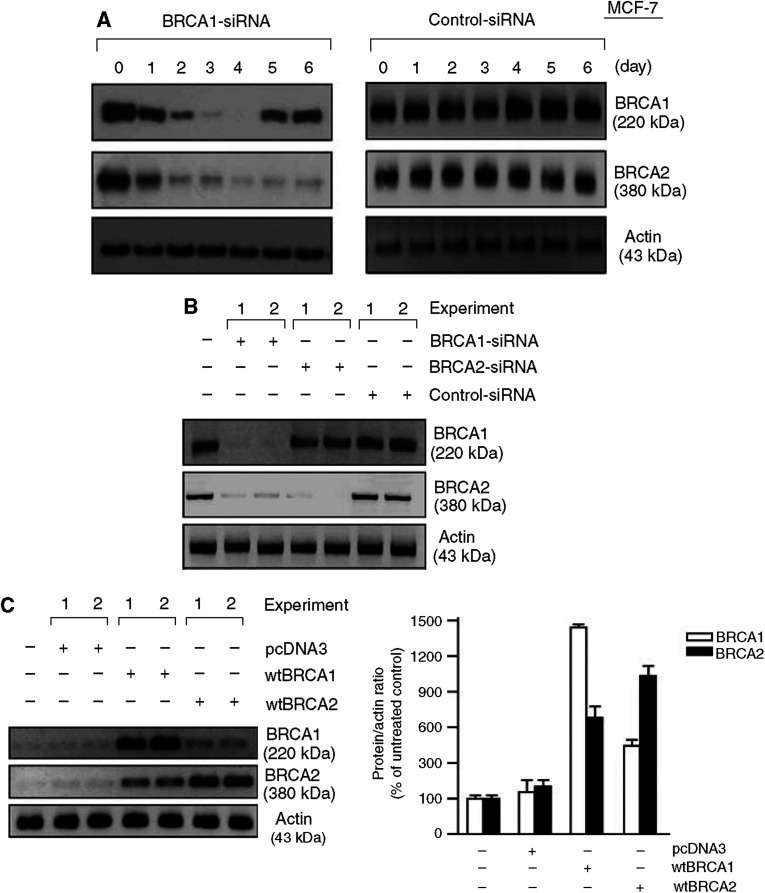

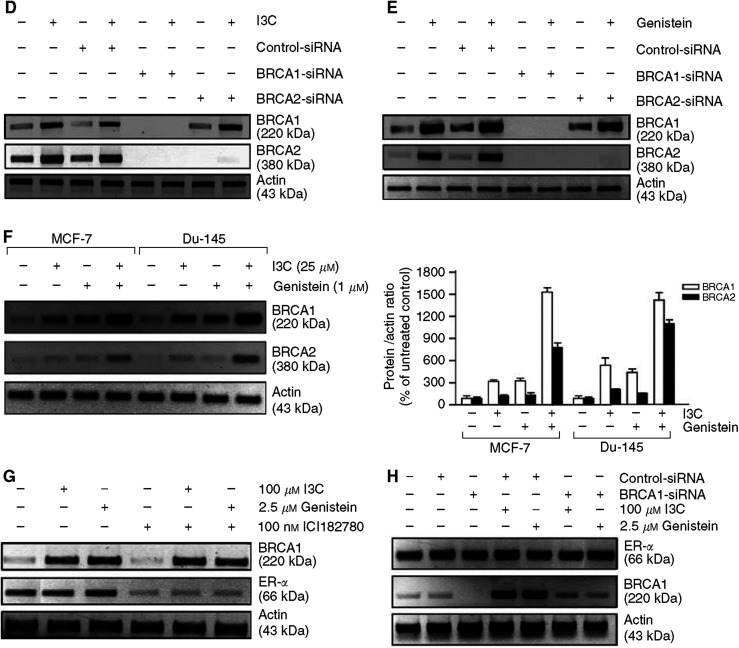
Dependence of phytochemical-induced expression of BRCA2 on BRCA1. (**A**) BRCA1-siRNA causes time-dependent loss of BRCA1 and BRCA2 in MCF-7 cells. Subconfluent proliferating cells were treated with BRCA1-siRNA (left) or control-siRNA (right) (50 nM) for different times, harvested, and Western blotted for BRCA1, BRCA2, and actin. (**B**) Effect of BRCA1-siRNA on BRCA2 protein levels and *vice versa* in DU-145 cells. Cells were treated with BRCA1, BRCA2, or control-siRNA (50 nM) for 72 h and Western blotted for BRCA1, BRCA2, and actin. Results are shown for two separate cell treatments and protein isolations on the same blot. (**C**) Effect of wtBRCA1 on BRCA2 protein levels and *vice versa* in DU-145 cells. Cells were transfected overnight with wtBRCA1, wtBRCA2, or empty pcDNA3 vector, washed, postincubated for 24 h to allow gene expression, harvested, and Western blotted for BRCA1, BRCA2, and actin. Results are shown for two separate cell treatments and protein isolations on the same blot. The densitometry values are means±ranges of two experiments. (**D**) Effect of BRCA1 and BRCA2 siRNAs on BRCA induction by I3C. DU-145 cells were preincubated with the indicated siRNA (50 nM × 72 h) or no siRNA (transfection reagent only), then treated with I3C (40 *μ*M) for 24 h, and then harvested for Western blotting. (**E**) Effect of BRCA1 and BRCA2 siRNA on induction of BRCA1 and BRCA2 by genistein. DU-145 cells were preincubated with the indicated siRNA (50 nM × 72 h), treated with genistein (5 *μ*M) for 24 h, and harvested for Western blotting as above. (**F**) Induction of BRCA1 and BRCA2 by a combination of I3C plus genistein. MCF-7 or DU-145 cells were treated with low doses of I3C (25 *μ*M) and/or genistein (1 *μ*M) for 24 h and harvested for Western blotting. The densitometry values are means±ranges of two independent experiments. (**G**) Effect of ICI182,780 on phytochemical induction of BRCA1 in MCF-7 cells. Proliferating cells were incubated with the indicated agents for 24 h and then harvested for Western blotting for BRCA1, ER-*α*, or actin. (**H**) Effect of BRCA1 knockdown and phytochemicals on ER-*α* protein levels. MCF-7 cells were pretreated with BRCA1 or control siRNA as described above, exposed to the indicated doses of I3C or genistein for 24 h, and then Western blotted for ER-*α*, BRCA1, or actin.

Several factors suggest that the phytochemical induction of BRCA2 is due, in part, to BRCA1. Thus, BRCA1 and BRCA2 are coordinately regulated during development and during cell cycle progression (Rajan *et al*, 1996, 1997); and exogenous BRCA1 upregulates expression of BRCA2 in cultured cells (Fan *et al*, 1998). BRCA1 and BRCA2 are also coregulated in response to DNA-damaging agents and other cytotoxins (Andres *et al*, 1998; Fan *et al*, 1999a). To determine the role of BRCA1 in the induction of BRCA2, we utilised specific BRCA1 and BRCA2 siRNAs. The BRCA1-siRNA was validated in a prior study (Xiong *et al*, 2003). As illustrated in Figure 4A, a single application of 50 nM of BRCA1-siRNA caused a loss of BRCA1 and BRCA2 proteins by 48–72 h, while a control-siRNA had no effect on BRCA1 or BRCA2 levels.

Consistent with these findings, BRCA1-siRNA caused loss of BRCA1 and BRCA2 proteins in DU-145 cells, but the converse was not true (Figure 4B). Thus, BRCA2-siRNA caused loss of BRCA2 protein, but BRCA1 was unaffected or only slightly decreased (Figure 4B). The control-siRNA had little or no effect on BRCA1 or BRCA2. In overexpression studies, a wild-type (wt) BRCA1 expression vector caused significant induction of BRCA2 levels; whereas wtBRCA2 caused a more modest rise in BRCA2 levels (Figure 4C). Next, we tested the role of BRCA1 in the induction of BRCA2 levels by I3C and by gensitein. In DU-145 cells pretreated with BRCA1-siRNA, there was little or no detectable BRCA2 in control, I3C-treated, or genistein-treated cells (Figure 4D and E, respectively). In cells treated with BRCA2-siRNA, BRCA2 was nearly undetectable in control and phytochemical-treated cells. However, BRCA1 was significantly induced by both I3C and genistein. A control-siRNA had no effect on the expression or induction of BRCA proteins. These findings suggest that the induction of BRCA2 by phytochemicals is dependent, in part, upon BRCA1, but not *vice versa*.

### Combination of I3C plus genistein

We tested the effects of a combination of low doses of I3C (25 *μ*M) and genistein (1 *μ*M) on BRCA protein levels in MCF-7 and DU-145 cells. The combination of (I3C+genistein) had a greater effect on BRCA induction than either agent alone (Figure 4G). These effects appeared to be supra-additive or synergistic for BRCA1 in MCF-7 cells and for BRCA2 in both cell types, although we did not perform studies using a range of different doses. Thus, 1 *μ*M of genistein by itself caused only a modest increase in BRCA2 levels; but the combination of (I3C+genistein) caused a much larger increase in BRCA2 levels than did I3C alone.

### Effect of inhibition of ER-*α* on BRCA1 expression

To determine if ER-*α* might have a role in the induction of BRCA1 by phytochemicals, MCF-7 cells were treated with I3C or genisetin in the absence or presence of ICI182,780 (Fulvestrant), an anti-oestrogen that causes degradation of ER-*α*. Here, ICI182,780 caused loss of ER-*α* protein but had no effect on the ability of I3C or genistein to induce BRCA1 protein (Figure 4G). As illustrated in Figure 4H, neither BRCA1-siRNA, nor I3C, nor genistein had ER-*α* protein levels in MCF-7 cells. Taken together with the findings that I3C and genistein can induce BRCA expression in ER-*α*-negative cell types (e.g., DU-145), these results suggest that the induction of BRCA expression by phytochemicals is not mediated through the oestrogen receptor.

### Role of BRCA1 in mediating some biological actions of I3C and genistein

While it is clear that I3C and genistein induce BRCA gene expression in human cancer cells, the roles of the BRCA genes in mediating the biologic responses to these phytochemicals is not established. We tested these roles utilising the BRCA1 and BRCA2 siRNA and other reagents.

#### Cytotoxicity

**Figure 5:**
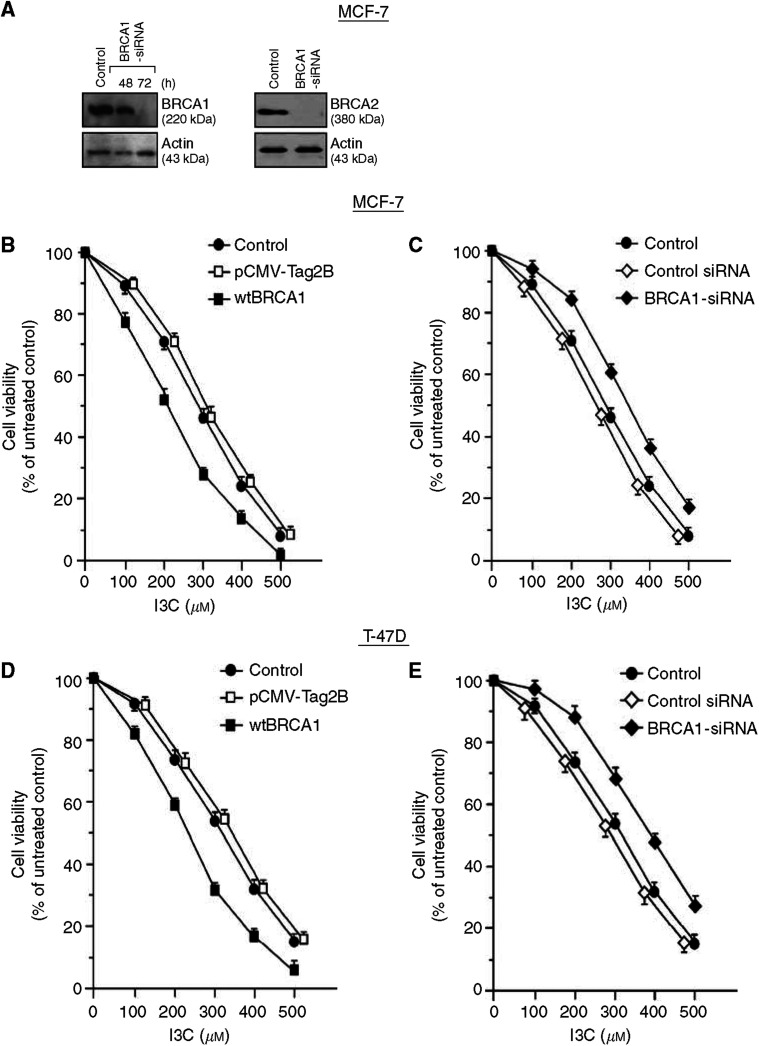

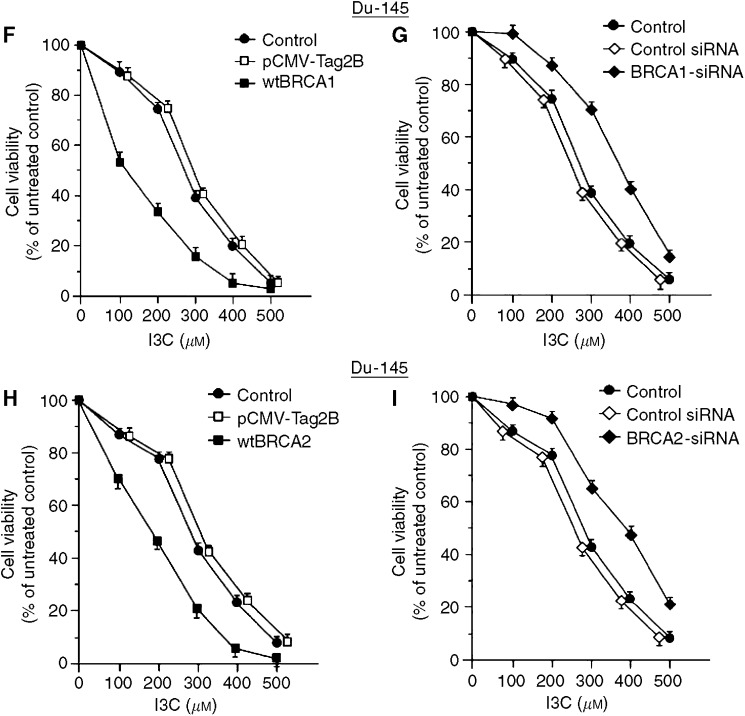
Contribution of BRCA1 and BRCA2 to I3C-mediated cytotoxicity. (**A**) The effects of BRCA1-siRNA (48 and 72 h) and BRCA2-siRNA (72 h) (50 nM) on BRCA1 and BRCA2 protein levels, respectively, in MCF-7 cells. MCF-7 (**B** and **C**), T47D (**D** and **E**), and DU-145 (**F** and **G**) cells were experimentally manipulated to increase (wtBRCA1) or decrease (BRCA1-siRNA) BRCA1 levels, treated with different doses of I3C, and tested for cell viability using MTT assays. In (**H**) and (**I**), DU-145 cells were manipulated to increase (wtBRCA2) or decrease (BRCA2-siRNA) BRCA2 levels, exposed to different doses of I3C, and tested for cell viability using MTT assays. *Methodology* (**B**–**I**). To increase BRCA1 levels, subconfluent cells in 96-well dishes were transfected with wtBRCA1 overnight (see Materials and Methods), washed, postincubated for 24 h, exposed to different doses of I3C for 24 h, and assayed for MTT dye reduction. To decrease BRCA1 levels, cells were pretreated with BRCA1- or control-siRNA (50 nM × 72 h) or mock-transfected (control) and assayed for sensitivity to I3C as above. For BRCA2 experiments, DU-145 cells were transfected with wtBRCA2 or treated with BRCA2- or control-siRNA (as above) and assayed as described above for sensitivity to I3C. Cell viability values are expressed relative to the 0 I3C control and are means±s.e.m.'s for 10 replicate wells. *Statistical comparisons*. Cell viability comparisons were made using two-tailed *t*-tests. Significant differences were as follows: MCF-7 wtBRCA1 *vs* control, *P*<0.001 at 100–400 *μ*M I3C; MCF-7 BRCA1-siRNA *vs* control, *P*<0.001 at 200–500 *μ*M I3C; T47D wtBRCA1 *vs* control, *P*<0.001 at 100–500 *μ*M I3C; T47D BRCA1-siRNA *vs* control, *P*<0.001 at 200–500 *μ*M I3C; DU-145 wtBRCA1 *vs* control, *P*<0.001 at 100–400 *μ*M I3C; DU-145 BRCA1-siRNA *vs* control, *P*<0.001 at 100–500 *μ*M I3C; DU-145 wtBRCA1 *vs* control, *P*<0.001, 100–400 *μ*M I3C; and DU-145 BRCA2-siRNA *vs* control, *P*<0.001 at 200–500 *μ*M I3C.

At high doses (⩾100 *μ*M), I3C causes cytotoxicity and apoptosis in cancer cell lines (Chen *et al*, 2001; Chinni *et al*, 2001; Rahman *et al*, 2003; Sarkar *et al*, 2003). Exogenous BRCA1 causes increased susceptibility to apoptosis due to DNA-damaging agents (e.g., adriamycin), although wtBRCA1 does not induce apoptosis by itself (Fan *et al*, 1998, 2001b). We tested the effect of manipulating BRCA levels on I3C cytotoxicity using MTT assays. Figure 5A shows the ability of BRCA1-siRNA and BRCA2-siRNA to reduce BRCA1 and BRCA2 protein levels, respectively, by 48–72 h. In three different cell lines (MCF-7, T47D, and DU-145), we found that BRCA1-siRNA protected cells against I3C cytotoxicity (Figure 5C, E and G). BRCA1-siRNA-treated cells usually showed survival rates about 15±5% higher than control-siRNA-treated cells (*P*<0.001, two-tailed *t*-tests), although the degree of protection by BRCA1-siRNA was greater for DU-145 cells at 300–400 *μ*M of I3C (21–32%).

In contrast, cells transfected with a wtBRCA1 gene were more sensitive to I3C than control cells (*P*<0.001) (Figure 5B, D, and F). At 200–400 *μ*M of I3C, MCF-7 and T47D cells transfected with wtBRCA1 showed survival rates about 15–20% lower than control-transfected cells; while wtBRCA1-transfected DU-145 cells showed 15–40% lower survival than control-transfected cells at 100–400 *μ*M I3C. These findings suggest that BRCA1 modulates the cytotoxicity of I3C and that endogenous BRCA1 contributes the cytotoxicity of I3C.

Since the BRCA1-siRNA downregulates BRCA2 levels, we tested the ability of BRCA2 to modulate sensitivity to high doses of I3C. As was true for BRCA1, a wtBRCA2 expression vector sensitised DU-145 cells to I3C (Figure 5H); while BRCA2-siRNA conferred decreased sensitivity to I3C (*P*<0.001 at all doses) (Figure 5I). Taken together with the previous results, these findings suggest that (1) both BRCA genes modulate cellular sensitivity to I3C and (2) the modulation of sensitivity to I3C by BRCA1 is due, in part, to alterations in BRCA2 levels.

#### Oestrogen receptor--*α* signalling

**Figure 6:**
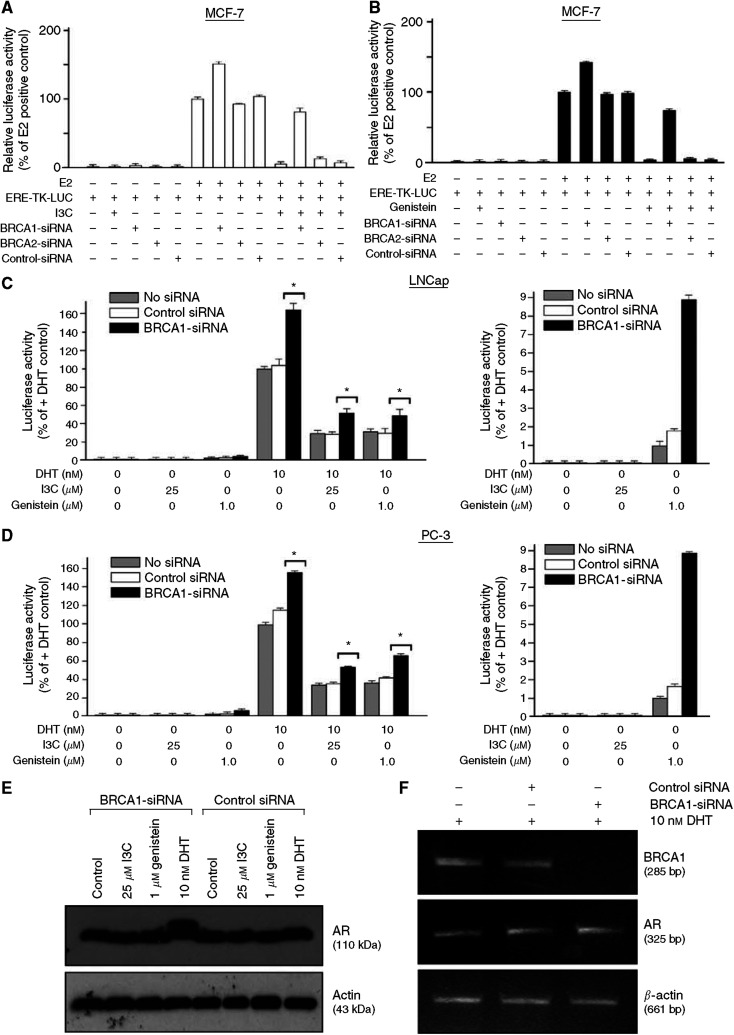
Contribution of BRCA genes to regulation of ER-*α* and AR activity by I3C and genistein. (**A**) Rescue of I3C inhibition of E2-stimulated ER-*α* activity by BRCA1-siRNA. MCF-7 cells were pretreated with BRCA1-siRNA, BRCA2-siRNA, control-siRNA (50 nM × 72 h), or no siRNA (vehicle only). After the first 48 h of siRNA treatment, they were transfected with the ERE-TK-Luc reporter overnight, washed, postincubated±17*β*-estradiol (E2, 1 *μ*M) and ±I3C (100 *μ*M) for 24 h, and tested for luciferase activity. Values are expressed relative to the +E2 positive control (no siRNA, no I3C) and are means±s.e.m.'s of three independent experiments. In each experiment, each assay condition was tested in four replicate wells, and the values were averaged. BRCA1 (but not BRCA2 or control) siRNA reversed the inhibition of E2-stimulated ER-*α* activity by I3C (*P*<0.001). (**B**) Rescue of genistein inhibition of E2-stimulated ER-*α* activity by BRCA1-siRNA. The experiment was performed as described above, except that the cells were treated ±genistein (5 *μ*M) instead of I3C. Luciferase values are expressed relative to the +E2 positive control (no siRNA, no genistein) and are means±s.e.m.'s of three independent experiments, with each assay condition tested in four replicate wells per experiment. BRCA1 (but not BRCA2 or control) siRNA reversed the inhibition of E2-stimulated ER-*α* activity by genistein (*P*<0.001). (**C**, **D**) Contribution of BRCA1 to inhibition of DHT-stimulated AR activity by I3C and genistein. LNCaP (**C**) or PC-3 (**D**) cells were pretreated with BRCA1-siRNA, control-siRNA (50 nM × 72 h), or no siRNA. LNCaP cells, which are AR-positive, were transfected with an androgen-responsive reporter (ARE-TK-Luc); while PC-3 cells, which are AR-negative, were cotransfected with an AR expression vector plus ARE-TK-Luc. The cells were treated with dihydrotestosterone (DHT, 10 nM), I3C (25 *μ*M), and/or genistein (1.0 *μ*M) for 24 h and assayed for luciferase activity. BRCA1-siRNA enhanced DHT-stimulated AR activity and partially rescued the inhibition of AR activity by I3C and genistein (left panels). The asterisks indicate a significant comparison (*P*<0.01). (**E**) Effect of BRCA1-siRNA and phytochemicals on AR protein levels in LNCaP cells. Cells were pretreated with BRCA1 or control siRNA and then treated with I3C, genistein, or DHT as described in (**C**). The cells were then harvested and Western blotted for AR or actin. (**F**) Effect of BRCA1-siRNA on AR mRNA levels. LNCaP cells were pretreated with BRCA1- or control-siRNA, treated with DHT for 24 h, and harvested for semiquantitative RT–PCR to detect BRCA1, AR, or *β*-actin.

We showed that I3C causes dose-dependent inhibition of estradiol (E2)-stimulated ER-*α* activity in cervical and breast cancer cells, by the use of an E2-responsive reporter (ERE-TK-Luc) and by testing the effect of I3C on expression of endogenous E2-responsive genes (Meng *et al*, 2000b). Since wtBRCA1 strongly inhibits ER-*α* signalling (Fan *et al*, 1999b), we hypothesised that BRCA1 might contribute to the inhibition of ER-*α* activity by I3C. Thus, we assayed the effects of BRCA siRNAs on the ability of I3C and genistein to inhibit E2-stimulated ER-*α* activity (Figure 6). While genistein is called a ‘phytoestrogen’ because it has weak oestrogenic activity in the absence of E2, it acts as an inhibitor of ER-*α* in the presence of E2. Thus, genistein caused dose-dependent inhibition of E2-stimulated ER-*α* activity in MCF-7 cells (data not shown). In this study, we did not observe pro-oestrogenic effects of genistein. However, we did not specifically test conditions that would elicit such effects.

Figure 6A shows the effect of pretreatment with BRCA siRNAs on inhibition of E2-stimulated ER-*α* activity by I3C. These data are the means±s.e.m.'s of three independent experiments. BRCA1 (but not BRCA2) siRNA caused a modest increase in E2-stimulated ER-*α* activity. Under conditions in which I3C caused >90% inhibition of ER-*α* activity, pretreatment with BRCA1-siRNA (but not BRCA2- or control-siRNA) substantially restored E2-stimulated ER-*α* activity (*P*<0.001, two-tailed *t*-test). Similar results were obtained using genistein (Figure 6B). Thus, BRCA1 (but not BRCA2 or control) siRNA significantly reversed the genistein-mediated inhibition of E2-stimulated ER-*α* activity (*P*<0.001). Previously, we identified an N-terminal fragment of the BRCA1 protein (amino acids 1–302) that functioned as a DN inhibitor of the full-length wild-type BRCA1 and ‘rescued’ the wtBRCA1-mediated inhibition of ER-*α* activity (Fan *et al*, 2001a, 2001b). Here, we found that transient transfection of a BRCA1 amino acid 1–302 expression vector (DN-BRCA1) partially rescued the inhibition of E2-stimulated ER-*α* activity by I3C alone, genistein alone, or the combination of I3C plus genistein (*P*<0.001) (data not shown). These findings suggest that the inhibition of E2-stimulated ER-*α* activity by I3C and genistein is due, in part, to BRCA1.

#### AR signalling

Both I3C and genistein can interact with and modulate the AR. Thus, I3C is an AR antagonist (Le *et al*, 2003), while genistein can activate the AR in the absence of ligand but inhibit DHT-induced AR activity (Maggiolini *et al*, 2002). We tested the role of BRCA1 in mediating the effects of low doses of I3C (25 *μ*M) or genistein (1 *μ*M) on AR signalling. We studied LNCaP cells (an androgen-responsive cell line) using their endogenous AR and PC-3 (an androgen-insensitive cell line) using transiently expressed wild-type AR. Consistent with published findings, genistein (but not I3C) caused a modest activation of an androgen-responsive reporter (ARE-TK-Luc) in the absence of DHT (Figure 6C and D, right panels), while both I3C and genistein caused inhibition of DHT-stimulated AR activity (*P*<0.001) (Figure 6C and D, *left* panels). Interestingly, pretreatment with BRCA1-siRNA caused an increase in DHT-stimulated AR activity (relative to control-siRNA or vehicle-treated cells) in the absence of I3C or genistein and partially rescued the inhibition of AR activity by I3C and genistein (*P*<0.01). Similar results were observed in LNCaP and PC-3 cells. Pretreatment with BRCA1-siRNA caused an increase in AR protein levels in cells treated with DHT, whereas BRCA1-siRNA by itself and control-siRNA in the absence or presence of DHT had little or no effect on AR levels (Figure 6E). In contrast, BRCA1-siRNA had no effect on AR mRNA levels in the presence of DHT (Figure 6F). The implications of these findings are considered in the Discussion.

### Role of endoplasmic reticulum stress in induction of BRCA expression

#### Agents that cause endoplasmic reticulum stress induce BRCA expression

**Figure 7:**
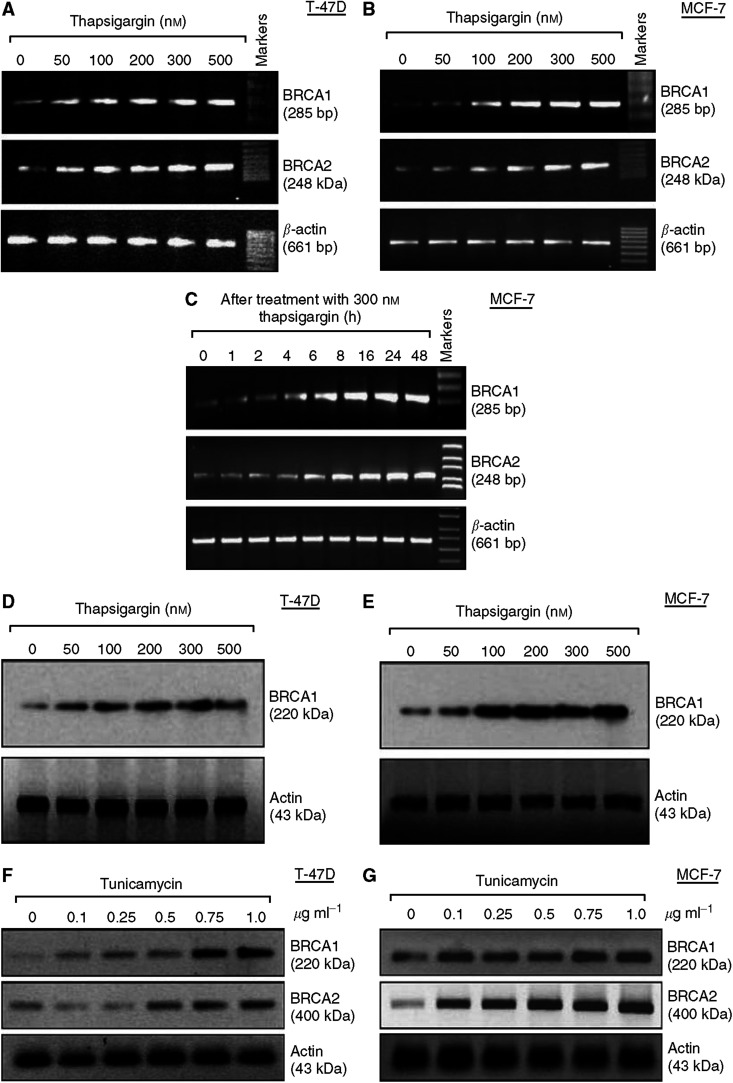
Thapsigargin and tunicamycin upregulate BRCA gene expression. (**A**, **B**) Dose–response for induction of BRCA mRNAs by thapsigargin in T47D (**A**) and MCF-7 (**B**) cells. Subconfluent proliferating cells were incubated with the indicated doses of thapsigargin for 24-h and then harvested for semiquantitative RT–PCR analysis of BRCA1, BRCA2, and *β*-actin (control gene). (**C**) Time course for induction of BRCA1 mRNAs by thapsigargin in MCF-7 cells. MCF-7 cells were incubated with thapsigargin (300 nM) for different time intervals up to 48-h and then harvested for semiquantitative RT–PCR analysis of BRCA1, BRCA2, and *β*-actin. (**D**, **E**) Dose–response for induction of BRCA1 protein by thapsigargin in T47D (**D**) and MCF-7 (**E**) cells. Cells were incubated with the indicated doses of thapsigargin for 24-h and then harvested for Western blotting to detect BRCA1 and actin (control for loading and transfer). (**F**, **G**) Dose–response for induction of BRCA proteins by tunicamycin in T47D (**F**) and MCF-7 (**G**) cells. Cells were incubated with the indicated doses of thapsigargin for 24-h and then harvested for Western blotting to detect BRCA1, BRCA2, and actin.

A recent study showed that DIM, a major metabolite of I3C, causes an endoplasmic reticulum-like stress response, similar to the unfolded protein response in yeast (Sun *et al*, 2004). Diindolylmethane caused phosphorylation of eucaryotic translation initiation factor 2*α* (EIF2A) linked to increased levels of ATF4 protein; activation of IRE1 (the homologue of inositol-requiring 1); a rapid increase in the stress-specific spliced form of XBP-1 mRNA; and induction of multiple stress-response genes, including CHOP (or GADD153), GADD34, GADD45A, XBP-1, GRP78, and GRP94 (Carter *et al*, 2002; Sun *et al*, 2004). Here, we tested the ability of two agents known to cause an endoplasmic reticulum stress response to induce BRCA expression. Incubation of T47D or MCF-7 cells with thapsigargin, a selective inhibitor of the endoplasmic reticulum Ca^+2^-dependent ATPase, caused dose- and time-dependent induction of BRCA1 and BRCA2 mRNA expression (Figure 7A–C). Induction of both BRCA genes occurred after a 24-h incubation with doses as low as 50–100 nM of thapsigargin (Figure 7A and B), while a time course study showed that BRCA1 mRNA was induced by thapsigargin (300 nM) after 4–6 h, with BRCA2 mRNA induction occurring slightly later (Figure 7C). MTT assays revealed no toxicity at thapsigargin doses ⩽300 nM (data not shown). Western blotting revealed induction of BRCA1 protein in T47D and MCF-7 cells at 50–100 nM of thapsigargin (Figure 7D and E).

We also saw dose-dependent induction of BRCA1 and BRCA2 proteins by tunicamycin (Figure 7F and G), an agent that causes an endoplasmic reticulum stress response by inhibition of protein glycosylation. These findings suggest that activation of an endoplasmic reticulum stress response by two distinct agents upregulates the expression of BRCA1 and BRCA2.

#### The ER stress-response kinase PERK is required for BRCA1 induction by I3C

**Figure 8:**
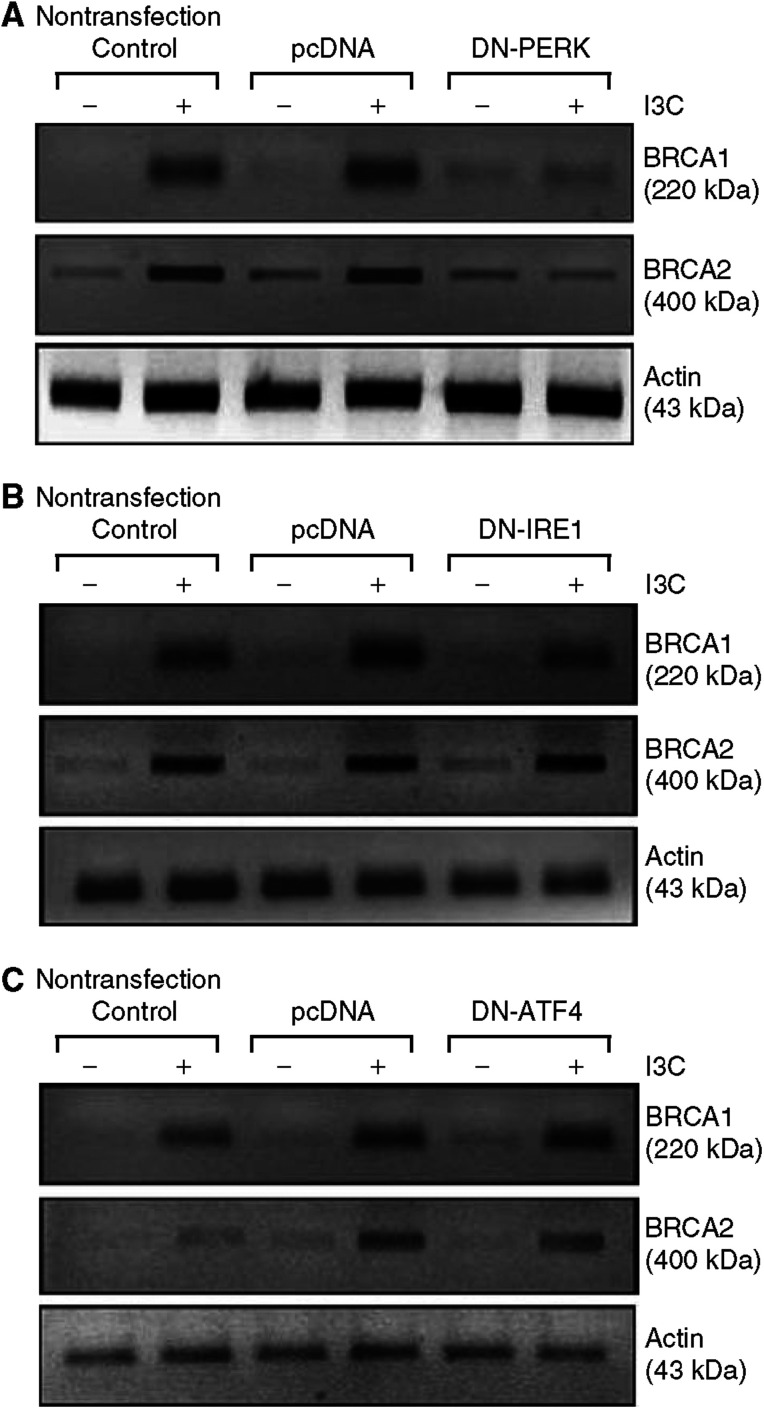
Induction of BRCA expression by I3C requires the kinase PERK (EIFAK3). (**A**) Dominant negative (DN) PERK blocks I3C induction of BRCA protein. Subconfluent proliferating MCF-7 cells were transfected overnight with control (vehicle only), empty pcDNA3 vector, or DN-PERK (15-*μ*g plasmid DNA per 100-mm dish) using Lipofectamine™. The transfected cells were washed, allowed to recover for several hours, treated±I3C for 24-h, and harvested for Western blotting to detect BRCA1, BRCA2, or actin (control for loading and transfer). (**B**) Dominant negative IRE1 fails to block I3C induction of BRCA protein. Assays were performed as described in (**A**), except using DN-IRE1 instead of DN-PERK. (**C**) Dominant negative ATF4 fails to block I3C induction of BRCA protein. Assays were performed as described in (**A**), except using DN-ATF4 instead of DN-PERK.

We tested the role of several endoplasmic reticulum stress signalling proteins in I3C induction of BRCA1 and BRCA2, using DN expression vectors. PERK (EIF2AK3) is a kinase that phosphorylates EIF2A in response to endoplasmic reticulum stress, causing inhibition of protein synthesis associated with the accumulation of ATF4 protein (Rutkowski and Kaufman, 2003). Transfection of a DN-PERK blocked the ability of I3C (100 *μ*M) to induce both BRCA1 and BRCA2 protein levels (Figure 8A). However, a DN-ATF4 vector failed to inhibit I3C-induced BRCA1 and BRCA2 expression (Figure 8B). Finally, we tested the role of IRE1 (the homolog of yeast inositol-requiring 1) – an endoplasmic reticulum membrane-localised kinase and endoribonuclease that is activated in response to stress (Shen *et al*, 2001) – in mediating BRCA induction. Here, a DN-IRE1 vector failed to inhibit I3C-induced BRCA1 or BRCA2 expression (Figure 8C). These findings suggest that PERK, an upstream component of endoplasmic reticulum and nutritional stress-response pathways, is required for BRCA induction.

#### I3C, genistein, and BRCA1 stimulate endoplasmic reticulum stress signalling

**Figure 9:**
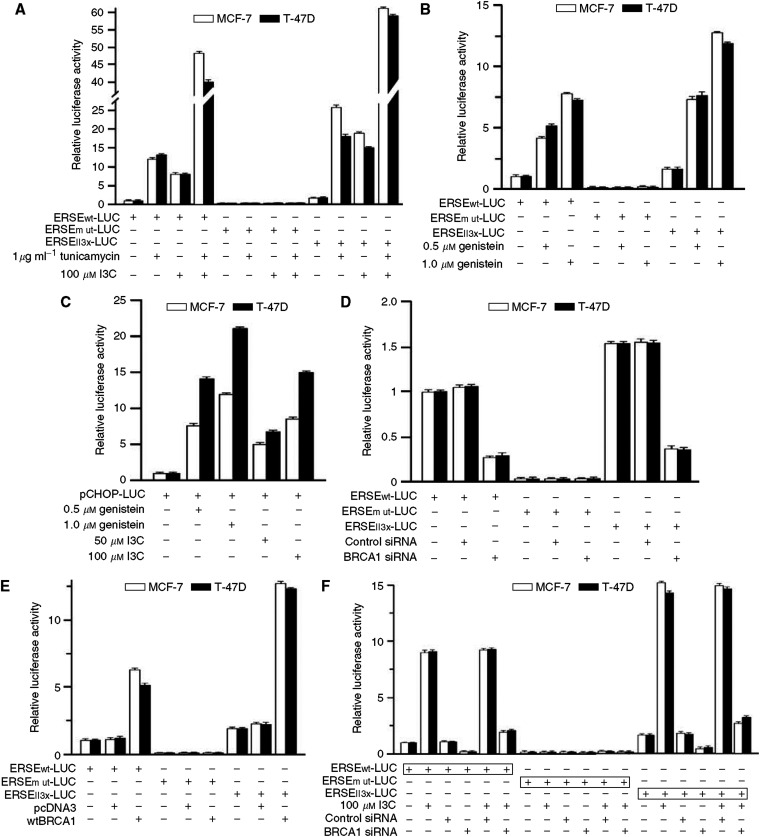

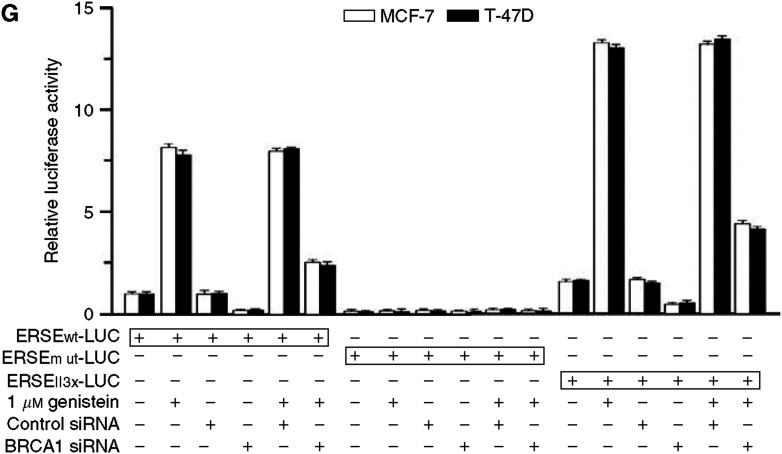
Stimulation of endoplasmic reticulum stress signalling by I3C, genistein, and BRCA1. (**A**) Stimulation of ERSE-driven reporter activity by I3C and tunicamycin. MCF-7 or T47D cells were transfected overnight with the indicated luciferase reporter, washed, postincubated with tunicamycin (1 *μ*g/ml) and/or I3C (100 *μ*M) for 24-h, and harvested for luciferase assays. Luciferase activity was expressed relative to that observed using the ERSEwt-Luc reporter in the absence of tunicamycin or I3C. Values are means±s.e.m.'s of four replicate wells. The reporters tested were driven by the wt endoplasmic reticulum stress-response element (ERSEwt-Luc), a mutant ERSE (ERSEmut-Luc), and three copies of the ERSE-II element (ERSEII3x-Luc). (**B**) Stimulation of ERSE reporter activity by genistein. Assays were performed as above, except that after transfection of reporters, the cells were treated with genistein (0.5 or 1.0 *μ*M) for 24 h. (**C**) Stimulation of CHOP promoter–reporter activity by I3C and gensitein. Cells were transfected overnight with the a reporter composed of the CHOP promoter upstream of a luciferase gene (pCHOP-Luc), washed, and postincubated with different doses of I3C or genistein for 24 h. Luciferase activity was expressed relative to that in the absence of I3C or genistein. The values are means±s.e.m.'s of four replicate wells. (**D**) Dependence of basal ERSE reporter activity on endogenous BRCA1. Proliferating cells were pretreated with BRCA1-siRNA, control-siRNA (50 nM × 72-h), or no siRNA (vehicle only). After 48 h of siRNA treatment, they were transfected with the indicated ERSE reporter. After siRNA treatment and transfection, the cells were washed, postincubated for 24 h, and harvested for luciferase assays. Values are expressed relative to the ERSEwt-Luc control (no siRNA) and are means±s.e.m.'s of four replicate wells. (**E**) Stimulation of ERSE reporter activity by wtBRCA1. Cells were cotransfected overnight with wtBRCA1, empty pcDNA3 vector, or no vector (vehicle only) and the indicated reporter. The cells were washed and postincubated for 24 h. Luciferase values are expressed relative to the ERSEwt-Luc reporter in the absence of wtBRCA1 or pcDNA3 and are means±s.e.m.' of quadruplicate wells. (**F**) Dependence of I3C-stimulated ERSE activity on endogenous BRCA1. Assays were performed as described in (**D**), except that after siRNA treatment and transfection, the cells were postincubated for 24 h in the absence or presence of I3C (100 *μ*M). (**G**) Dependence of genistein-stimulated ERSE activity on endogenous BRCA1. Assays were performed as described in (**F**), except using genistein (1 *μ*M) instead of I3C.

Endoplasmic reticulum stress signalling is mediated, in part, by transcription via *cis*-acting DNA elements in target genes, including the endoplasmic reticulum stress-response element (ERSE), unfolded protein response element (UPRE), and a second type of ERSE (ERSE-II) (Yamamoto *et al*, 2004). We tested the ability of I3C, genistein, and BRCA1 to stimulate reporters driven by the wild-type ERSE (ERSEwt-Luc), a mutant ERSE (ERSEmut-Luc, a negative control), and the ERSE-II element (ERSEII3x-Luc). Figure 9A shows the ability of I3C and/or tunicamycin to induce ERSE signalling in T47D and MCF-7 cells. Each agent caused a significant induction of ERSEwt and ERSE-II activity (*P*<0.001), but the ERSEmut-Luc reporter showed little or no activity. Similarly, low doses of genistein (0.5 and 1.0 *μ*M) significantly activated the ERSEwt and ERSE-II but not the ERSEmut reporter (*P*<0.001) (Figure 9B). CHOP is a stress-responsive transcriptional regulator implicated in apoptosis due to severely impaired endoplasmic reticulum function (Oyadomari and Mori, 2004). Indole-3-carbinol and genistein caused dose-dependent activation of a CHOP-Luc reporter in T47D and MCF-7 cells (*P*<0.001) (Figure 9C).

We next found that BRCA1 (but not control) siRNA inhibited basal ERSEwt and ERSE-II reporter activity (*P*<0.001) (Figure 9D), whereas wtBRCA1 (but not empty pcDNA3 vector) enhanced basal ERSEwt and ERSE-II signalling (*P*<0.001) Figure 9E). Pretreatment with BRCA1-siRNA severely attenuated the ability of I3C to upregulate ERSEwt and ERSE-II reporter activity in T47D and MCF-7 cells (*P*<0.001) (Figure 9F). Finally, pretreatment with BRCA1 (but not control) siRNA, blocked the ability of genistein to stimulate ERSEwt and ERSE-II activity (*P*<0.001) (Figure 9G). These findings suggest that both I3C and genistein stimulate ERSE activity and BRCA1 is required for I3C- and genistein-induced ERSE activity.

## DISCUSSION

We showed that two phytochemicals with potential cancer prevention activity, I3C and genistein, each upregulate expression of the BRCA1 and BRCA2 breast cancer genes. Significant increases in BRCA1 and BRCA2 protein levels (>2-fold) were observed at relatively low doses of these agents (20 *μ*M of I3C and 0.5–1 *μ*M of genistein), suggesting potential physiologic relevance of these findings. While I3C and genistein have a variety of cellular actions *in vitro* and protect against various types of cancer *in vivo*, it is unclear which of these actions is essential for cancer prevention and to what degree. BRCA1 and BRCA2 have been identified as tumour suppressors for several different hormone-responsive cancer types (breast and prostate cancers (BRCA1 and BRCA2) and endometrial and cervical cancer (BRCA1)) and a non-endocrine cancer type (pancreas cancer (BRCA1 and BRCA2)) (Struewing *et al*, 1997; Thompson and Easton, 2002; Aretini *et al*, 2003; Edwards *et al*, 2003). Thus, it is plausible to hypothesise that some of the chemoprevention activity of I3C and genistein is due to stimulation of BRCA expression.

Evidence of a role for BRCA1 in sporadic mammary carcinogenesis supports this idea. Thus, BRCA1 expression is decreased or absent in a significant proportion of sporadic breast and ovarian cancer cases, in part because of hypermethylation of the BRCA1 promoter on CpG islands (Wilson *et al*, 1999; Rice *et al*, 2000). A significant fraction of sporadic breast cancers (46%) were found to be haploinsufficient for BRCA1 (Staff *et al*, 2003). Moreover, several agents that may contribute to breast cancer development (polycyclic aromatic hydrocarbons (e.g., benzo(a)pyrene) and alcohol) downregulate BRCA1 expression (Fan *et al*, 2000; Jeffy *et al*, 2002). While the loss of BRCA1 expression or function is linked to breast cancer, the role of upregulation of either BRCA gene in preventing cancer is unclear. Several studies suggest that BRCA expression is strongly induced in proliferating cells undergoing differentiation, including those in the mammary gland during puberty and pregnancy (Rajan *et al*, 1996, 1997; Bernard-Gallon *et al*, 2001). These findings suggest that the BRCA genes are highly induced and play anticarcinogenic roles at specific times during mammary development. If so, then agents that upregulate BRCA1 and BRCA2 in mammary epithelial cells may prevent cancer development.

Genistein has multiple biologic actions, some of which may contribute to its cancer prevention activity: (1) inhibition of hormone-dependent and -independent cancer cell proliferation, antiapoptotic signalling (NF-*κ*B and c-Akt), toposomerase II*α* activity, tyrosine kinase activity, and angiogenesis; and (2) stimulation of TGF-*β* signal transduction, p53 and Chk2 kinase activity, antioxidant activity, and differentiation (Castle and Thrasher, 2002; Sarkar and Li, 2002). Our findings indicate that genistein upregulates BRCA1 and BRCA2 expression in breast and prostate cancer cell lines. In a previous study, genistein caused an increase in BRCA2 mRNA (but not protein) levels in MDA-MB-231 and MCF-10A but not in MCF-7 cells (Vissac-Sabatier *et al*, 2003). In that study, daidzein, another soy isoflavone, had no effect on BRCA2 expression. There were methodologic differences between that study and ours (e.g., the use of affinity chromatography to measure BRCA2 protein). Nonetheless, the reason for the differences with our results are unclear, since we found that the BRCA2 protein was highly induced in a reproducible fashion in four different human cancer cell lines.

Several factors suggest that BRCA induction by phytochemicals is not due to ER-*α* signalling: (1) the BRCA genes were induced equally by I3C and genisein in MCF-7 cells treated without *vs* with ICI182,780 (which causes degradation of ER-*α*); (2) another anti-oestrogen, Tamoxifen, had no effect on BRCA1 levels (Jones *et al*, 2005); (3) BRCA1 and BRCA2 were strongly induced by I3C and genistein in ER-*α*-negative DU-145 prostate cancer cells; and (4) I3C upregulates BRCA1 expression in ER-*α* negative breast cancer (MDA-MB-231 and MDA-MB-468) and cervical cancer cells or human foreskin keratinocytes (Meng *et al*, 2000a 2000b, 2001; Carter *et al*, 2002). Finally, our results suggest induction of BRCA expression by an oestrogen-independent mechanism involving an endoplasmic reticulum stress-like response.

We showed some activities of I3C and genistein are blocked or partially reversed by inhibition of BRCA1: (1) upregulation of BRCA2 by I3C or genistein; (2) cytotoxicity due to high doses of I3C; (3) inhibition of E2-stimulated ER-*α* activity by I3C and/or genistein in breast cancer cells; and (4) inhibition of DHT-stimulated AR activity by I3C and genistein in prostate cancer cells. Studies using under- and overexpression of BRCA1 and BRCA2 suggest that the ability of BRCA1 to modulate I3C cytotoxicity is due, in part, to BRCA2. On the other hand, the I3C inhibition of ER-*α* activity is due mostly to BRCA1, and not BRCA2. Indole-3carbinol and genistein may exert BRCA1-dependent and BRCA1-independent effects on ER-*α* and AR signalling, since BRCA1-siRNA can influence ER-*α* and AR activity in the absence of phytochemicals. It is also noteworthy that some of the effects of genistein on oestrogen receptor signalling may be mediated through ER-*β*, since genistein has a higher affinity for ER-*β* than for ER-*α* (Kuiper *et al*, 1998).

Rescue of the inhibition of ER-*α* by I3C and/or genistein is consistent with previous studies showing that wtBRCA1 inhibits ER-*α* signalling and a DN-BRCA1 rescues the inhibition (Fan *et al*, 1999b; 2001a, 2001b). However, the finding that BRCA1-siRNA stimulates AR activity and partially reverses the inhibition of DHT-induced AR activity by I3C and genistein is somewhat surprising, since wtBRCA1 and wtBRCA2 were reported to stimulate AR signalling (Yeh *et al*, 2000; Shin and Verma, 2003). Presently, we do not have a good explanation for this discrepancy; but we have observed that wtBRCA1 either has no effect on or modestly enhances DHT-stimulated activation of several ARE-driven reporters (ARE-TK-Luc and MMTV-Luc) (unpublished results), suggesting that the consequences of underexpressing BRCA1 may not be predictable based on overexpression models. Interestingly, treatment of LNCaP cells with BRCA1-siRNA plus DHT caused an increase in AR protein, which may have contributed to the increase in DHT-stimulated AR activity in cells treated with BRCA1-siRNA. The increase in AR protein was not due to an increase in mRNA but was associated with a slight decrease in electrophoretic mobility, consistent with post-translation modification (e.g., phosphorylation). Since the cofactor environment is critical to AR activity, it is also possible that loss of BRCA1 causes upregulation of an AR coactivator(s) and/or downregulation of an AR corepressor(s).

In a recent study, it was reported that prepubertal exposure of rats (age 7–20 days) to genistein caused prolonged upregulation of BRCA1 mRNA expression in the mammary glands (Cabanes *et al*, 2004). The induction of BRCA1 was associated with morphologic evidence of mammary differentiation and a reduced susceptibility to 7,12-dimethylbenz[a]anthracene-induced mammary tumours. These findings suggest that BRCA1 may contribute to protection against mammary tumorigenesis by genistein, but cause and effect have not yet been proven.

Taken together, our findings suggest that BRCA1 and BRCA2 are potential molecular targets for the chemoprevention agents I3C and genistein. Although we did not extensively study combinations of agents, our findings suggest that I3C and genistein may act synergistically or supra-additively to stimulate BRCA1 and BRCA2 expression. Further work is required to determine if BRCA1 and BRCA2 are potential intermediate biomarkers that predict the efficacy of these or other prevention agents and whether a combination of I3C and genistein can provide greater cancer prevention efficacy with the same or less toxicity (i.e., a better therapeutic ratio).

Our studies suggest that I3C induces BRCA expression via an endoplasmic reticulum stress-like pathway, although this hypothesis needs to be definitively proved. A previous study revealed that the I3C metabolite DIM activates multiple endoplasmic reticulum stress pathways, leading to induction of downstream target genes (e.g., chaperones) and activation of c-Jun N-terminal kinases JNK1 and JNK2 (Sun *et al*, 2004). Here, we found that I3C activates ERSE and ERSE-II signalling and induces CHOP promoter activity in T47D and MCF-7 cells, consistent with an endoplasmic reticulum stress-like response. Importantly, two agents known to cause endoplasmic reticulum stress, thapsigargin and tunicamycin, also induced BRCA1 and BRCA2 expression; and a DN PERK, an upstream component of endoplasmic and nutritional stress-response pathways, blocked I3C-induced BRCA1 expression.

The mechanism(s) by which endoplasmic reticulum signalling induces BRCA1 expression remains to be determined. We analysed the BRCA1 promoter sequence (GeneBank Accession U37574) for canonical ERSE (ccaat-n9-ccacg), UPRE (tgacgtgg/a), and ERSE-II(attgg-n-ccacg) elements (where *n*=any nucleotide). The 1.5-kb region upstream of the BRCA1-coding sequence did not contain any complete ERSE or ERSE-II elements, but it did contain a number of half-sites. However, this region does contain two possible UPREs: tgacgtga and tggcgtgg. The role of these elements in mediating phytochemical induction of BRCA1 remains to be determined.

Our results also suggest a role for BRCA1 in endoplasmic reticulum stress signalling, since BRCA1 was found to positively regulate ERSE and ERSE-II activity. Thus, knockdown of BRCA1 inhibited basal ERSE and ERSE-II activity, while overexpression of BRCA1 stimulated ERSE and ERSE-II activity; and knockdown of BRCA1 reduced the I3C-induced ERSE and ERSE-II activity. These results are consistent with a model in which BRCA1 is both a target and a mediator of the endoplasmic reticulum stress response, as are other transcriptional regulators, such as activating transcription factor 6 (ATF6) and X box-binding protein 1 (XBP1). Finally, we found that low doses of genistein (0.5–1.0 *μ*M) significantly induced ERSE, ERSE-II, and CHOP promoter activity, suggesting that genistein can activate endoplasmic reticulum stress response signalling, although the extent of the pathway activated and its significance remain to be determined. The ability of genistein to induce endoplasmic reticulum stress response signalling could, in part, explain how low doses of genistein induce BRCA gene expression.
